# Stomatal development in the context of epidermal tissues

**DOI:** 10.1093/aob/mcab052

**Published:** 2021-04-20

**Authors:** Keiko U Torii

**Affiliations:** 1 Howard Hughes Medical Institute and Department of Molecular Biosciences, The University of Texas at Austin, AustinTX, USA; 2 Institute of Transformative Biomolecules (WPI-ITbM), Nagoya University, Nagoya, Aichi, Japan

**Keywords:** Stomata, peptide hormones, auxin, receptor kinase, signal transduction, bHLH proteins, meristemoid, stomatal-lineage ground cell, guard cell, pavement cell, trichome, hydathode water pore, hypocotyl epidermal cell files, root hair patterning

## Abstract

**Background:**

Stomata are adjustable pores on the surface of plant shoots for efficient gas exchange and water control. The presence of stomata is essential for plant growth and survival, and the evolution of stomata is considered as a key developmental innovation of the land plants, allowing colonization on land from aquatic environments some 450 million years ago. In the past two decades, molecular genetic studies using the model plant *Arabidopsis thaliana* identified key genes and signalling modules that regulate stomatal development: master regulatory transcription factors that orchestrate cell state transitions and peptide–receptor signal transduction pathways, which, together, enforce proper patterning of stomata within the epidermis. Studies in diverse plant species, ranging from bryophytes to angiosperm grasses, have begun to unravel the conservation and uniqueness of the core modules in stomatal development.

**Scope:**

Here, I review the mechanisms of stomatal development in the context of epidermal tissue patterning. First, I introduce the core regulatory mechanisms of stomatal patterning and differentiation in the model species *A. thaliana*. Subsequently, experimental evidence is presented supporting the idea that different cell types within the leaf epidermis, namely stomata, hydathodes pores, pavement cells and trichomes, either share developmental origins or mutually influence each other’s gene regulatory circuits during development. Emphasis is placed on extrinsic and intrinsic signals regulating the balance between stomata and pavement cells, specifically by controlling the fate of stomatal-lineage ground cells (SLGCs) to remain within the stomatal cell lineage or differentiate into pavement cells. Finally, I discuss the influence of intertissue layer communication between the epidermis and underlying mesophyll/vascular tissues on stomatal differentiation. Understanding the dynamic behaviours of stomatal precursor cells and their differentiation in the broader context of tissue and organ development may help design plants tailored for optimal growth and productivity in specific agricultural applications and a changing environment.

## Introduction

Stomata, turgor-driven cellular valves on the plant aerial epidermis, serve as an interface between a plant and its environment. By dynamically adjusting stomatal pore apertures, stomata facilitate gas exchange for photosynthesis while minimizing water loss via transpiration. The acquisition of both stomata and epidermal pavement cells that protect plants from dry atmospheric conditions are considered key developmental innovations that enabled green plants to conquer the land some 450 million years ago (Peterson *et al.*, 2010; Chater *et al.*, 2017). Stomata exist in land plants including basal land plants, with the exception of liverworts (Chater *et al.*, 2016; Bowman *et al.*, 2017). Increasing evidence suggests that the core genes and signalling mechanisms governing stomatal development, which were discovered in the model angiosperm *Arabidopsis thaliana*, are conserved across the land plant species, with unique variations signifying diversity in morphology and function of stomatal complexes (Chater *et al.*, 2017; Nunes *et al.*, 2020). A corollary to this hypothesis is a striking finding from the genome of an aquatic grass plant, Eelgrass *Zostera marina*, which revealed that the loss of genes regulating stomatal development and epicuticular wax synthesis signifies the adaptation of the land-based angiosperm to the sea (Olsen *et al.*, 2016). The evolutionary history of land plants and their adaptation to aquatic environments imply that development of stomata and epidermal pavement cells is coupled to generate functional epidermis. However, studies of stomatal development, including the influence of environmental and hormonal signals, have so far been focused within the context of cell lineages, rather than at a tissue level (Lau and Bergmann, 2012; Qi *et al.*, 2020).

**Fig. 1.**
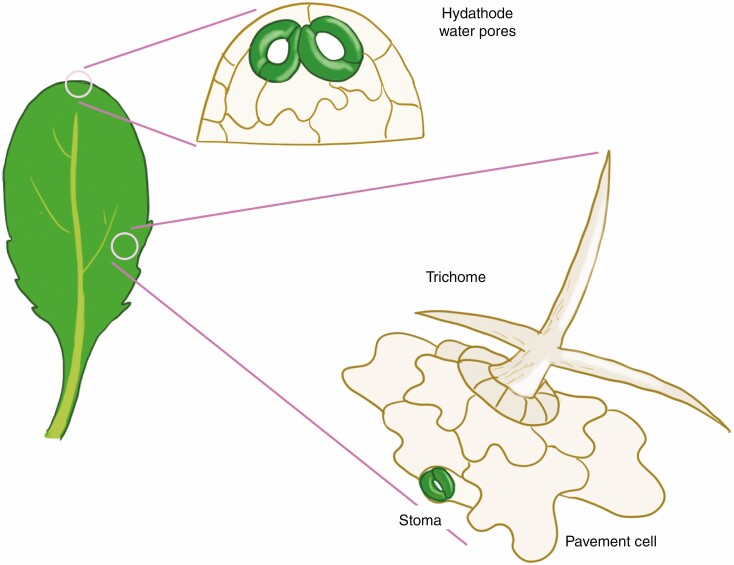
Epidermal cell types in arabidopsis rosette leaf. A schematic diagram of an arabidopsis rosette leaf (left). Hydathode water pores are found at/near the tip of the leaf edges and are typically paired large stomata that remain open (top right). Epidermal pavement cells, trichomes and stomata (right) are found on the leaf blade throughout. Trichomes are only found on the adaxial side of the leaf blade, whereas more stomata are located on the abaxial side. Neither stomata nor trichomes differentiate from the epidermis immediately above the midvein.

In addition to stomata, the leaf epidermis of arabidopsis differentiates three additional primary cell types, epidermal pavement cells, trichomes and hydathode water pores, each responsible for specific functions (Fig. 1). Interlocking jigsaw puzzle-shaped pavement cells not only provide protection from numerous environmental insults, but also guide plant growth through localized elongation and expansion (Javelle *et al.*, 2011). Trichomes are branched, rigid hair-like structures that provide protection against herbivores (Schellmann and Hulskamp, 2005). Lastly, hydathode water pores located at the leaf margin function as a site of guttation to release water pressure (Cerutti *et al.*, 2019). We are beginning to understand how differentiation programmes of these different cell types are mutually influenced and co-ordinated at the level of gene regulatory circuits and cell–cell signalling. In this review, I first describe the basics of arabidopsis stomatal development, and explain the core genes and regulators of stomatal differentiation and patterning. In addition, some variations on a theme of stomatal development in distant plant species are introduced. Subsequently, I describe the core regulatory mechanisms underpinning the development of hydathode water pores, trichomes and pavement cells, and discuss how the core molecular mechanisms of stomatal development are integrated with other epidermal cell differentiation programmes. Specific emphasis will be given to stomatal-lineage ground cells (SLGCs), bipotent cells that can produce a stoma or pavement cell. Lastly, further roles of intertissue layer communication will be discussed. Through synthesis, I aim to bridge the gap in our knowledge of stomatal development in the broader context of tissues and organs, toward gaining a holistic view of its importance for plant growth and sustenance.

## THE CORE MECHANISMS OF STOMATAL DEVELOPMENT

### Stomatal cell lineages

**Fig. 2.**
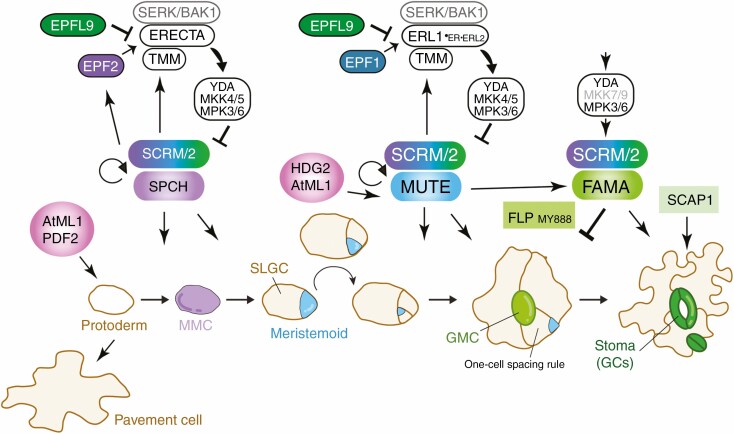
Core regulatory mechanism of stomatal development. In the typical dicot plant, arabidopsis, protodermal fate is specified by two HD-ZIP IV transcription factors, AtML1 and PDF2. Stomatal development initiates on a sub-set of protodermal cells termed meristemoid mother cells (MMCs: lilac) with high accumulation of the bHLH heterodimer module of SPCH and SCRMs (SPCH–SCRMs). An MMC undergoes an asymmetric entry division and gives rise to two daughter cells, meristemoid (cyan) and stomatal-lineage ground cell (SLGC: ivory). The meristemoids reiterate asymmetric amplifying division, which is maintained by high SPCH activity. A switch from the proliferation to differentiation state is regulated by the MUTE–SCRM module. MUTE promotes differentiation of the guard mother cell (GMC), and orchestrates the single symmetric cell division. A FAMA–SCRM bHLH module and Myb proteins FLP and MYB88 restrict the single symmetric cell division. FAMA and SCAP1 promote guard cell (GC) morphogenesis. Peptide–receptor kinase signalling pathways mediated by the EPF2–ERECTA–TMM and EPF1–ERL1–TMM ligand–receptor module restricts the entry into stomatal cell lineages and enforces proper asymmetric spacing division, respectively. EPFL9 (Stomagen) antagonizes the signalling by competitively binding to the receptors. The activated receptor associates with SERK/BAK1 co-receptor, and the signal is transduced via an MAPK cascade consisting of YODA–MKK4/5–MPK3/6. MPK3/6 is recruited by SCRM to downregulate SPCH and probably MUTE. The stomatal bHLH proteins directly regulate the cell–cell signalling components, and this negative feedback loop maintains proper cell fate specification.

Stomatal development has been extensively studied in arabidopsis, where a series of cell divisions and cell state transitions occur in a stereotypical manner to produce anisocytic stomatal complexes consisting of a two-cell stoma surrounded by up to three non-stomatal epidermal pavement cells (Nadeau and Sack, 2002*a*) (Fig. 2). Stomatal development initiates within a sub-population of protodermal cells, adopting an identity termed meristemoid mother cells (MMCs), which then initiate asymmetric division (asymmetric ‘entry’ division) that produces two daughter cells with different characteristics: a meristemoid cell and an SLGC (Fig. 2). The stem-like meristemoid reiterates asymmetric divisions (asymmetric ‘amplifying’ divisions), renewing itself while amplifying SLGCs. The SLGC can then re-adopt the MMC identity to give rise to a satellite meristemoid or, alternatively, terminally differentiate into a pavement cell (Fig. 2). The meristemoid eventually differentiates into a round guard mother cell (GMC), which subsequently divides symmetrically and terminally differentiates into a pair of guard cells enclosing a pore (Bergmann and Sack, 2007; Lau and Bergmann, 2012; Pillitteri and Torii, 2012; Herrmann and Torii, 2021) (Fig. 2).

### Transcription factor control of stomatal differentiation

Key molecular–genetic studies over the past two decades have unravelled core regulatory mechanisms of stomatal development (Bergmann and Sack, 2007; Lau and Bergmann, 2012; Pillitteri and Torii, 2012; Herrmann and Torii, 2021). Cell state transitions leading to stomatal differentiation are directed by sequential actions of three basic–helix–loop (bHLH) proteins, SPEECHLESS (SPCH), MUTE and FAMA, as well as their heterodimeric partners, SCREAM (SCRM; also known as ICE1) and SCRM2, here referred to as SCRMs (Ohashi-Ito and Bergmann, 2006; MacAlister *et al.*, 2007; Pillitteri *et al.*, 2007; Kanaoka *et al.*, 2008) (Fig. 2). The activity of SPCH–SCRMs in the nucleus drives the initiation of stomatal cell lineages and promotes asymmetric amplifying divisions of meristemoids and MMCs; *spch* or *scrm scrm2* double mutants confer an epidermis solely composed of (non-stomatal) pavement cells. Next, MUTE–SCRMs orchestrate the cell state switch in meristemoids from proliferation to differentiation into GMCs (Pillitteri *et al.*, 2007; Kanaoka *et al.*, 2008) (Fig. 2). FAMA–SCRMs regulate the symmetric division of GMCs and trigger their terminal differentiation into guard cells. In concert with FAMA, the Myb protein FOUR LIPS (FLP) and its redundant paralogue MYB88 restrict the symmetric division of the GMC (Ohashi-Ito and Bergmann, 2006; Kanaoka *et al.*, 2008) (Fig. 2). Recent experimental evidence and modelling studies have shown that MUTE orchestrates a single symmetric division of GMCs by directly inducing the expression of cell cycle regulators to initiate division, as well as FAMA and FLP, which directly repress the cell cycle regulators to end the division cycle (Lai *et al.*, 2005). Additional transcription factors, including a C2H2 Dof transcription factor *STOMATA CARPENTER1* (*SCAP1*), are required for proper guard cell maturation (Negi *et al.*, 2013) (Fig. 2).

### Cell–cell signalling enforcing stomatal patterning

Stomatal mechanics rely on rapid water and ion movement between stomatal guard cells and neighbouring non-stomatal epidermal cells. Stomata are patterned in such a way that two stomata will not form immediately adjacent to each other (Nadeau and Sack, 2002*a*). This probably reflects the underlying physiology for optimal solute exchange between the stomatal guard cells and neighbouring non-stomatal cells (such as subsidiary cells) for efficient stomatal movement as well as for effective water vapour and carbon dioxide diffusion through stomata (Roth-Nebelsick, 2007). This so-called ‘one-cell spacing rule’ is enforced by local peptide–receptor kinase signalling. In arabidopsis, a family of secreted cysteine-rich peptides EPIDERMAL PATTERNING FACTORS (EPF)/EPF-LIKES (EPFL) act as paracrine signals to enforce proper stomatal patterning (Hara *et al.*, 2007; Hunt and Gray, 2009; Rychel *et al.*, 2010) (Fig. 2). Initial spatial patterning of stomatal progenitor cells is controlled by EPF2, which is secreted from MMCs and inhibits neighbouring cells from adopting stomatal lineage identity. EPF2 peptide is primarily perceived by ERECTA-family receptor kinases and its partner TOO MANY MOUTHS (TMMs) (Nadeau and Sack, 2002*b*; Shpak *et al.*, 2005; Hara *et al.*, 2009; Hunt and Gray, 2009; Lee *et al*., 2012, 2015). Upon ligand perception, the ERECTA–TMM receptor complex with co-receptor SOMATIC EMBRYOGENESIS RECEPTOR-LIKE KINASES (SERKs)/BAK1 trigger activation of a mitogen-activated protein kinase (MAPK) signalling cascade composed of YODA MAPKKK, MKK4/5 MAPKKs and MPK3/6 MAPKs (Bergmann *et al.*, 2004; Wang *et al.*, 2007; Bemis *et al.*, 2013; Meng *et al.*, 2015) (Fig. 2). *EPF2* is directly induced by SPCH–SCRMs, and in turn EPF2 signalling leads to inhibition of SPCH–SCRMs via MPK3/6-dependent phosphorylation and degradation (Lampard *et al.*, 2008; Lau *et al.*, 2014; Horst *et al.*, 2015; Putarjunan *et al.*, 2019) (Fig. 2). Differentiation of meristemoids as well as the enforcement of the ‘one-cell spacing rule’ are mediated by EPF1, which is primarily perceived by ERECTA-LIKE1 (ERL1), forming a receptor complex with TMM and SERKs/BAK1 (Lee *et al.*, 2012; Meng *et al.*, 2015). EPF1 signalling inhibits MUTE activity, while MUTE–SCRM modules, in turn, directly induce *ERL1* and *TMM* gene expression, thereby forming a negative feedback loop to balance signal strengths (Qi *et al.*, 2017; Han *et al.*, 2018) (Fig. 2).

### Conservation of core regulators of stomatal development

Thus far, research in other plant species strongly supports the hypothesis that the core mechanisms of stomatal development are conserved throughout land plants that possess stomata. For example, recent studies showed that in the moss *Physcomitrella patens*, which generates a single row of stomata on its sporophyte (Peterson *et al.*, 2010), ancient *SPCH-MUTE-FAMA* (*PpSMF*) bHLH genes as well as *EPF1-TMM* (and probably *ERECTA*) peptide receptor genes (*PpEPF1*, *PpERECTA* and *PpTMM*) regulate stomatal development and patterning (Chater *et al.*, 2016; Caine *et al.*, 2020). Likewise, the differentiation of highly derived and efficient stomatal complexes in monocot cereal species is regulated by the same conserved set of stomatal bHLH proteins and cell–cell signalling components, with some acquiring unique novel functions (Liu *et al.*, 2009; Raissig *et al*., 2016, 2017). The most striking example is *Brachypodium* MUTE (BdMUTE), which acquired a novel function to promote subsidiary cell development (Raissig *et al.*, 2017). Unlike *BdMUTE*, the loss-of-function mutation in *MUTE* (*OsMUTE*) confers excessive asymmetric division of a meristemoid and its eventual arrest, a phenotype equivalent to the arabidopsis *mute* mutant (Wu *et al.*, 2019). Instead of *OsMUTE*, *OsFAMA* appears to influence subsidiary cell division (Pillitteri *et al.*, 2007; Wu *et al.*, 2019), emphasizing that the precise functions of stomatal bHLHs may be co-opted differently amongst the grass species. Among the orthologues of cell–cell signalling components, EPFs and YODA MAPKKK exhibit clear functions in grass family stomatal development, including in wheat, barley and *Brachypodium* (Hughes *et al.*, 2017; Abrash *et al.*, 2018; Caine *et al.*, 2019; Dunn *et al.*, 2019). On the other hand, specific roles for the peptide receptors, including the ERECTA family, TMM and SERKs/BAK1, in stomatal patterning have not been demonstrated in grass species. Excellent review articles on grass stomatal development are available for further reading (Hepworth *et al.*, 2018; McKown and Bergmann, 2020; Nunes *et al.*, 2020).

## STOMATAL DEVELOPMENT IN THE CONTEXT OF EPIDERMAL PATTERNING

### Role of protoderms in stomatal development

Whereas the core mechanism of stomatal development has been extensively studied, stomatal development in leaves occurs in the context of epidermal tissues and, more broadly, the whole leaf. In addition to stomata, specialized cell types in the leaf epidermis, namely epidermal pavement cells, trichomes and hydathode water pores, all originate from protodermal cells. The protodermal fate is specified by the family of homeodomain-leucine zipper IV (HD-ZIP IV) proteins, AtML1 and its paralogue PDF2 (Lu *et al.*, 1996; Abe *et al.*, 2003) (Fig. 2). These transcription factors are sufficient to direct stomata and trichome differentiation in leaf cells of non-protodermal origin. For instance, ectopic *AtML1* overexpression triggers the differentiation of aberrant trichomes and stomata in internal mesophyll tissues (Takada *et al.*, 2013). During stomatal development, the HD-ZIP IV protein HDG2 (and, to a lesser extent, AtML1) is expressed in the meristemoids and promotes stomatal differentiation via upregulating *MUTE* expression (Fig. 2). Consistently, ectopic overexpression of *HDG2* confers ectopic stomata (but not ectopic trichomes) in the internal mesophyll tissues (Peterson *et al.*, 2013). It is still not clear how HD-ZIP IV proteins trigger the initiation of different leaf epidermal cell types or whether the regulatory programme driving the differentiation of a single epidermal cell type interferes (or co-operates) with any other. Recent studies are shedding light on the relationships between the core stomatal development mechanisms and those of other epidermal cell types.

### Stomata and hydathode water pores

The hydathode is a special organ on the leaf tips responsible for guttation – releasing water droplets from the vasculature to reduce hydrostatic pressure (Cerutti *et al.*, 2019). It is composed of vasculature, small parenchyma cells called epithum, and water pores from which the water droplets (dew drops) are released. The water pores are considered to be modified stomata: closely resembling stomata but with some notable differences. Unlike regular stomata, which rarely form next to another stoma (due to the ‘one-cell spacing rule), the water pores in arabidopsis are composed of a pair of large stomata in contact, and which remain constitutively open (Pillitteri *et al.*, 2008) (Fig. 2). The water pores in the grass species, such as *Brachypodium*, rice and maize, are aligned along the leaf tip, tend to remain open and seemingly lack proper subsidiary cells (Jauneau *et al.*, 2020).

Although the specific molecular mechanisms generating hydathode water pores have not yet been explored, at least one of the canonical stomatal transcription factor genes, *MUTE*, is required for water pore differentiation, since water pores are absent in *mute* cotyledons and leaves (Pillitteri *et al.*, 2008). It has been proposed that hydathode differentiation occurs at the site of auxin synthesis during leaf morphogenesis (Aloni, 2001). Consistent with this hypothesis, the initial expression of the auxin response reporter *DR5::GFP* is visible at the leaf tip where initial *MUTE* expression is also detected. Whether the differentiation of water pores requires high levels of auxin is an interesting question. Whereas *mute* cotyledons and leaves lack water pores, they still exhibit high activity of the auxin reporter *DR5:GFP* at the leaf tips. Likewise, treatment with the auxin transport inhibitor *N*-1-naphthylphthalamic acid (NPA) did not disrupt *MUTE* promoter activity at the leaf tip (Pillitteri *et al.*, 2008). Thus, the tip-localized auxin may act independently from *MUTE* or, alternatively, auxin promotes the water pore initiation before *MUTE* acts (e.g. via *SPCH*). Very recently, RNA-seq analysis of Arabidopsis hydathode regions was performed in an attempt to identify genes specifically expressed in hydathodes, which include several auxin biosynthesis and auxin-related genes (Yagi *et al.*, 2020). Such an analysis may reveal genes and pathways uniquely associated with water pore differentiation and additional overlapping features with stomata development.

### Patterning of stomata and trichomes

**Fig. 3.**
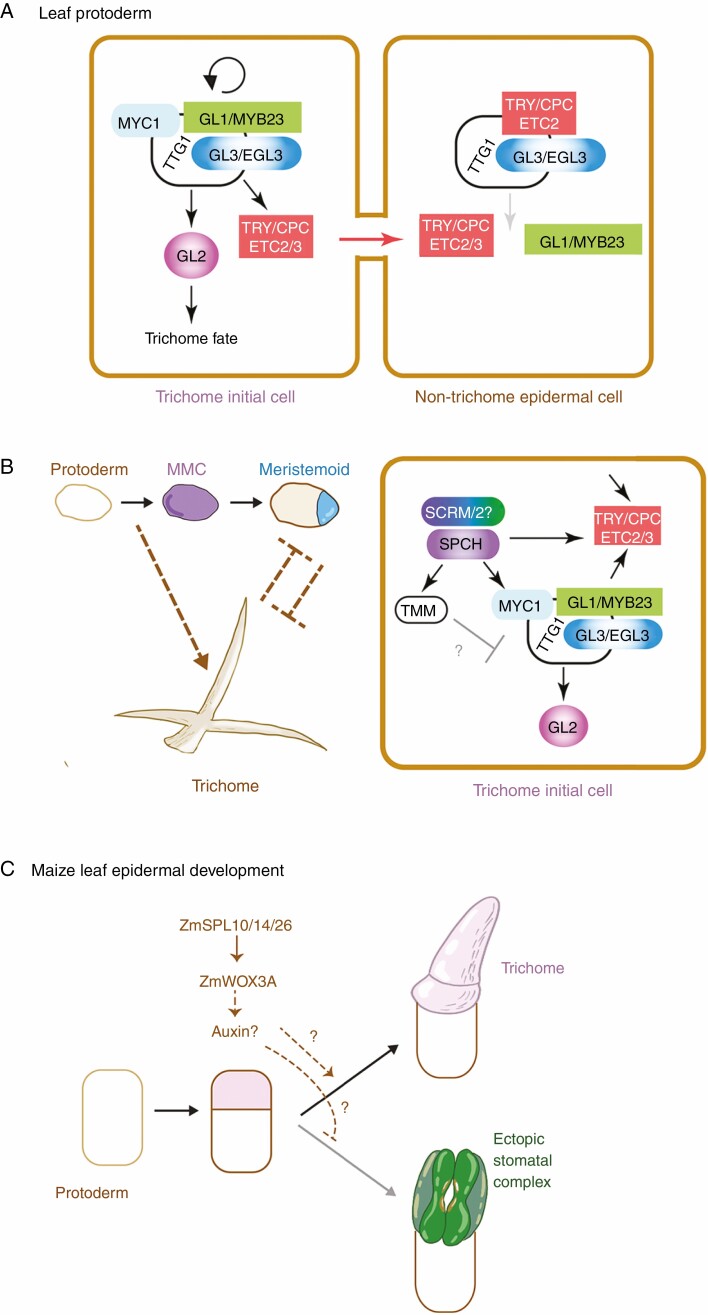
Trichome initial patterning and its intersection with the core stomatal developmental programme. (A) Trichome initial fate specification. The Myb–bHLH–WD40 complex containing GL1/MYB23, GL3/EGL3 (and MYC2) and TTG autoactivates itself and induces the expression of the HD-ZIP IV protein GL2, which specifies the trichome fate. The Myb–bHLH–WD40 complex in addition induces the expression of small Myb proteins, TRY/CPC/ETC2/ETC3, which can move from cell to cell via plasmodesmata and repress the activity of the Myb–bHLH–WD40 in the adjacent cell through competitive inhibition. (B) Role of the core stomatal development programme in trichome fate specification. Left: protoderm and MMCs may induce the trichome initiation programme, whereas later in the developmental progression both stomata and trichome precursors may mutually exclude each other’s fate. Right: in the protoderm/MMC, SPCH directly upregulates *MYC1*. In addition, SPCH directly upregulates *TMM*, whose overexpression reduces trichome numbers via an unknown mechanism. (C) Regulation of trichome (hair) development in maize. A trichome precursor cell (pink) is generated via an asymmetric cell division of a protodermal cell (white). ZmSPL10/14/26 upregulate the expression of *ZmWOX3A*, and promote trichome fate of the precursor, probably through auxin. In the absence of these ZmSPL genes, a stomatal complex transdifferentiates from the precursor instead of the trichome. The figure is modified and re-drawn from Kong *et al.* (2021).

Yet another specialized cell type on the leaf epidermis is the trichome, which serves as a mechanical protection against herbivores (Mauricio and Rausher, 1997) among other functions, such as production of essential oils and other metabolic products in glandular trichomes (Glas *et al.*, 2012; Huchelmann *et al.*, 2017). In arabidopsis, a trichome is an extremely enlarged, polarized unicellular non-glandular structure formed on the adaxial side of the leaf (Schellmann and Hulskamp, 2005). Like stomata, leaf trichomes are formed *de novo* in an evenly distributed manner, and clonal analysis has shown that cell–cell communication also specifies the trichome initials. However, the 2-D spatial patterns of stomata and trichome distribution are governed by distinct molecular and signalling modules. Trichome fate is specified by a transcription factor complex consisting of a MYBR2R3 protein, GLABRA1 (GL1), a bHLH protein, GLABRA3 (GL3), and its paralogues ENHANCER OF GL3 (EGL3) and MYC1, as well a WD40-repeat protein, TRANSPARENT TESTA GL1 (TTG1) (Larkin *et al.*, 1994; Walker *et al.*, 1999; Payne *et al.*, 2000; Schellmann and Hulskamp, 2005; Zhao *et al.*, 2012) (Fig. 3A). The Myb–bHLH–WD40 transcription factor complex induces GL2, an HD-ZIP transcription factor directing trichome differentiation (Rerie *et al.*, 1994). The 2-D spatial patterns of trichome initial cells are maintained by lateral inhibition, which involves a family of short MYBR3 proteins, TRIPTYCON (TRY) CAPRICE (CPC), and redundant paralogues, ENHANCER OF TRY AND CPC2/3 (ETC2/3) (Schellmann *et al.*, 2002) (Fig. 3A). These short MBR3 proteins associate with GL3/EGL3 but fail to bind to the target promoter regions. The GL1–GL3/EGL3–TTG1 complex induces *TRY/CPC* expression, which is capable of moving to the neighbouring cells via plasmodesmata and inhibiting the activity of GL1–GFL3/EGL3–TTG1 by competitively displacing the transcriptional activator GL1 (Fig. 3A). This switches the Myb–bHLH–WD40 complex from an activator to a repressor of trichome fate (Schellmann and Hulskamp, 2005).

Phenotypic analyses of stomatal mutant and transgenic plants suggest complex regulatory relationships between stomatal development and trichome patterning. For example, the *scrm-D* mutant, in which SCRM is constitutively active, overproduces stomata seemingly at the expense of trichomes (Kanaoka *et al.*, 2008). Likewise, overexpression of *TMM* reduces trichome numbers and branching (Yan *et al.*, 2014), suggesting that stomatal development is antagonistic to trichome formation (Fig. 3B). Interestingly, however, a study of direct SPCH targets hints that the story is not that simple (Adrian *et al.*, 2015). The SPCH protein binds to the promoter region of *MYC1* and *ETC3*, and upregulates their transcripts, indicating that SPCH directly activates the genes promoting trichome differentiation (Fig. 3B). In addition, *ETC2* is strongly expressed in stomata and stomatal lineages, although loss-of-function *etc2* mutant as well as *try cpc etc2* triple mutant cotyledons exhibit no defects in stomatal density and patterning (Kirik *et al.*, 2004). These seemingly counterintuitive findings imply that the molecular mechanisms underlying the initiation of stomatal cell lineages also contribute to the initiation of trichomes. However, a rosette leaf of the *spch* null mutant produces trichomes in apparent proper density (Hara *et al.*, 2009), indicating that *SPCH* is not required for the initiation or spacing of trichomes. Thus, the biological significance of SPCH activation of trichome genes, such as *MYC1* and *ETC3*, remains unclear.

A very recent study of leaf epidermal development in maize revealed an antagonistic regulatory mechanism between trichome and stomatal development. Maize triple loss-of-function mutants lacking three *SQUAMOSA-PROMOTER BINDING PROTEIN-LIKE* (*SPL*) genes *ZmSPL10/14/26* develop glabrous leaf epidermis devoid of trichomes (Kong *et al.*, 2021). In maize, three different types of trichomes (known as macrohairs, prickle hairs and bicellular hairs) exist, but *Zmspl10/14/16* triple mutants lack all types of trichomes, but instead transdifferentiate ectopic stomatal complexes. This striking observation suggest that both maize trichomes and stomatal complexes originate from the same precursor cell (generated after the asymmetric division of a protodermal cell), and *ZmSPL10/14/26* are required to suppress stomatal fate (Kong *et al.*, 2021) (Fig. 3C). Through a molecular analysis, Kong *et al.* (2021) propose that *ZmSPL10/14/26* activates the expression of *ZmWOX3A*, which then activates an auxin biosynthesis gene. Perhaps, through the action of auxin, the stomatal precursor fate is suppressed to ensure proper trichome differentiation (Fig. 3C). Whether this pathway is maize specific or more broadly applicable to other species remains to be seen. In any event, the studies in arabidopsis and maize highlight the complex regulatory relationships between the development of stomata and trichomes.

### Stomatal patterning in hypocotyls: co-option of root hair patterning programmes

**Fig. 4.**
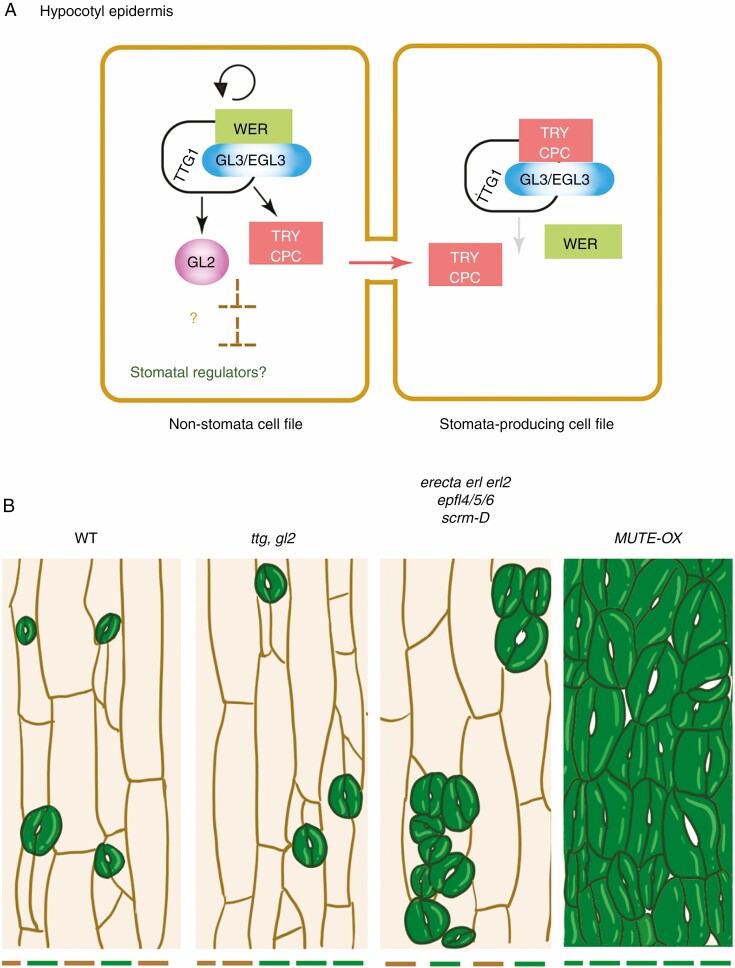
Hypocotyl epidermal patterning and its role in stomatal development. (A) The hypocotyl cell file follows the root hair/non-root hair epidermal patterning programme. Similar to the trichome fate specification programme, the Myb–bHLH–WD40 transcriptional complex consisting of WER–GL3/EGL3–TTG1 directly upregulates *GL2* expression, which specifies a non-root hair cell file and non-stomatal cell file in the root and hypocotyl epidermis, respectively. The Myb–bHLH–WD40 complex in addition induces inhibitory small Myb proteins, TRY/CPC. TRY/CPC move from cell to cell and repress the Myb–bHLH–WD40 complex by competitively displacing WER, and this leads to eventual differentiation to a root hair cell file and a stomata-producing cell file in root and hypocotyl epidermis, respectively. (B) Hypocotyl epidermis phenotypes. Wild-type (WT) hypocotyl produces ordered columns of stomata-producing (green bars) and non-stomata-producing (brown bars) cell files. The loss-of-function mutants in Myb–bHLH–WD40, such as *ttg* and *gl2* mutants, disrupt cell files. Core stomatal development mutants, such as *erecta erl1 erl2*, *epfl4/5/6* and *scrm-D*, produce stomatal clusters only within the stomata-producing cell files. In contrast, the *MUTE* overexpressor produces hypocotyls solely composed of stomata. These ectopic stomata are very large and align in files, suggestive of direct reprogramming of protodermal cells into stomata.

Unlike in cotyledons and leaves, the hypocotyl epidermis of arabidopsis shares characteristics of the root epidermis, with a common set of regulatory modules used in trichome development and root epidermal patterning (Schiefelbein, 2003; Grebe, 2012). During the development of the root epidermis, the Myb–bHLH–WD40 complex composed of WEREWOLF (WER)–GL3/EGL3–TTG1 induces *GL2* expression, which subsequently suppresses expression of a series of root hair initiation and elongation genes, including RHD6, to specify non-hair cell fate. *CPC/TRY* induced by the WER–GL3/EGL3–TTG1 complex moves to a neighbouring cell and outcompetes WER for binding with GL3/EGL3 to inhibit *GL2* induction, thereby promoting root hair cell fate (Wada *et al.*, 1997; Lee and Schiefelbein, 1999; Schiefelbein *et al.*, 2009) (Fig. 4A). The hypocotyl epidermis produces elongated, non-stomatal cell files and sunken stomata-producing files with cell division capacity. These cells exhibit no *GL2* expression. However, not all cells in stomata-producing cell files in the hypocotyl epidermis develop into stomata, indicating that the stomatal cell fate determination requires additional signals. The elongated non-stomata-producing cell files exhibit strong *GL2* expression, therefore sharing molecular characteristics of non-hair-producing root epidermal cell files (Berger *et al.*, 1998; Hung *et al.*, 1998) (Fig. 4A). Further mutant analyses showed that loss-of-function *gl2* and *ttg* mutants ectopically expand stomata-producing cell files in hypocotyls, resulting in ectopic stomata (Fig. 4B). Unlike *GL2* and *TTG*, *GL1* and *TRY* play a role only in the trichome patterning. Loss-of-function *gl1* and *try* mutants did not confer any effects on stomatal patterning in hypocotyls (Berger *et al.*, 1998; Hung *et al.*, 1998). Taken together, the hypocotyl stomatal patterning follows the root hair patterning rule.

Now that the underlying molecular players that potentiate the hypocotyl epidermis to make stomata have been elucidated, the important next question is to address their regulatory relationships with the core stomatal transcription factors (e.g. SPCH, MUTE, FAMA and SCRMs) and cell–cell signalling modules (e.g. EPF–ERECTA/TMM–MAPK). Indeed, phenotypic analysis demonstrated that the core regulators of stomatal development operate only in the ‘pre-patterned’ stomata-producing cell files: *erecta erl1 erl2*, *epfl4/epfl5/epfl6* (also known as *challah/chll1/chll2*) and *scrm-D* mutants all produce dense stomatal clusters in hypocotyls, but only within the stomata-producing cell files (Kanaoka *et al.*, 2008; Pillitteri *et al.*, 2008; Abrash *et al.*, 2011) (Fig. 4B). Likewise, *mute* mutant hypocotyls produce arrested meristemoids only in the stomata-producing cell files, but additional *gl2* or *ttg* mutations confer production of ectopic cell files with meristemoids (Pillitteri *et al.*, 2008).

Can the ectopic activation of stomatal development over-ride the hypocotyl cell file positional information? Indeed, ectopic overexpression of *MUTE* converted all hypocotyl epidermal cells into stomata, regardless of the original cell files (Pillitteri *et al.*, 2008). The large size and block wall-like arrangements of these ectopic stomata in *MUTE* overexpressors suggest that all hypocotyl epidermal cells underwent direct reprogramming to differentiate stomata (Pillitteri *et al.*, 2008) (Fig. 4B). Despite this observation, the molecular connection of the Myb–bHLH–WD40 (WER–GL3/EGL3–TTG1) transcription factor complex to the core stomatal development programme is not known. One possibility is that the transcription factor complex or its downstream effector, *GL2*, represses *SPCH* expression. Thus far, however, ChIP on chip experiments have not identified SPCH as a direct target of GL1 or GL3 (Morohashi and Grotewold, 2009). Very recently, the C2H2-Zinc Finger protein ZP1 was identified as a negative regulator of root hair development (Han *et al.*, 2020). ZP1 acts downstream of GL2 and directly represses the expression of a set of ‘root hair’ *bHLH* genes: *ROOT HAIR DEFECTIVE 6* (*RHD6*), which initiates root hair formation, and subsequently *RHD6-LIKE2* (*RSL2*) and *RSL4*, which promote root hair elongation (Han *et al.*, 2020). By analogy, it is tempting to speculate that a similar gene regulatory network may specify non-stomatal cell files in hypocotyls. Alternatively, the elongated and protruded non-stomatal-producing (and *GL2*-positive) cell files may harbour a specific epigenetic state that is incompatible with stomatal differentiation programmes. In this regard, it is worth mentioning that certain regulators of histone modifications, specifically HISTONE DEACETYLASE18 (HDA18), a negative regulator of histone acetylation, and GEM, a negative regulator of histone H3 K9 trimethylation (H3K9me3), are required for proper *GL2* expression and proper patterning of root hair cell files (Xu *et al.*, 2005; Caro *et al.*, 2007). Future studies may reveal the exact molecular connection of gene regulatory networks specifying hypocotyl epidermal patterning and stomatal development.

### Relationships between epidermal pavement cells and stomatal cell lineages

In addition to protecting plants from numerous environmental stresses, epidermal pavement cells provide a mechanical strength for plant growth and help guide organ morphogenesis (Bemis and Torii, 2007; Savaldi-Goldstein *et al.*, 2007). The arabidopsis epidermal pavement cells have been characterized extensively for their unique jigsaw-puzzle shapes with characteristic lobes and sinuses. Numerous empirical and mathematical modelling studies have been performed to address how pavement cell shapes emerge and how mechanical stress influences the pavement cell geometry through dynamic behaviours of microtubules, cytoskeletons and cell walls (Sampathkumar *et al.*, 2014; Armour *et al.*, 2015; Elsner *et al.*, 2018; Altartouri *et al.*, 2019; Haas *et al.*, 2020). Moreover, the plant hormone auxin co-ordinates pavement cell interdigitation via specific activation of two antagonistic small GTPase proteins: ROP2/4 determines the site of lobe expansion via local F-actin accumulation and, conversely, ROP6 specifies the site of the sinus via cortical microtubule organization through localized bundling (Fu *et al*., 2002, 2009; Xu *et al.*, 2010). The auxin efflux carrier PIN1 is implicated in orchestrating the process by supplying auxin flow (Xu *et al.*, 2010), although there is a contradictory observation refuting the role of PIN proteins in pavement cell morphogenesis (Belteton *et al.*, 2018).

The leaf epidermal pavement cell differentiation does not require other epidermal cell types, including stomatal cell lineages. The most extreme example is the *spch* mutant, which produces an epidermis composed solely of perfectly interdigitated pavement cells (MacAlister *et al.*, 2007; Pillitteri *et al.*, 2007). In fact, researchers took advantage of the *spch* mutant to describe and model the pavement cell morphogenesis without the confounding effects of ‘irregularly shaped’ stomatal lineage cells (Carter *et al.*, 2017). Although pavement cells can differentiate directly from protodermal cells, it is estimated that the vast majority of pavement cells in wild-type epidermis are derived from the stomatal lineage (approx. 67 % of all pavement cells in cotyledons and approx. 48 % in leaves) through the proliferative activities of MMCs and meristemoids (Geisler *et al.*, 2000). Thus, stomatal lineage cells not only produce stomata, but are also a major source for pavement cells.

The key cell type supplying new pavement cells is the SLGC, which is defined as the larger daughter cell generated by the asymmetric division of an MMC or meristemoid (Shpak *et al.*, 2005). The SLGC has interesting bi-potency: it could re-enter the stomatal initial state (MMC state) and undergo secondary asymmetric cell division to generate a satellite stoma, or it could lose the asymmetric cell division potential and differentiate into a pavement cell. Maintenance of the MMC state requires high amounts of SPCH–SCRMs (Horst *et al.*, 2015) and, as such, the mechanisms leading to the loss of expression of SPCH–SCRMs drive the eventual differentiation of SLGCs into pavement cells. In arabidopsis, both extrinsic EPF peptide signalling and intrinsic polarity pathways downregulate SPCH–SCRMs via the activation of MAPK cascades (Herrmann and Torii, 2021). Indeed, a tissue mosaic study using Cre–Lox recombination-dependent EPF1 overexpression sectors showed that secreted extrinsic signals influence adjacent cells as well as those within a range of a few cells (Zeng *et al.*, 2020). The intrinsic polarity component, BASL (BREAKING OF ASSYMMETRY IN THE STOMATAL LINEAGE), specifies the SLGC fate to one of the two daughter cells generated by the asymmetric cell division of a meristemoid (Dong *et al.*, 2009). In newly formed SLGCs, BASL localizes at the cellular cortex and recruits YODA, and in return MPK3/6 phosphorylates BASL in the nucleus (Zhang *et al.*, 2015). This BASL–MAPK positive feedback loop ensures the inhibition of SPCH accumulation, thereby preventing the SLGC from becoming a stomatal precursor cell (Zhang *et al.*, 2016).

SLGCs that are transitioning into pavement cells begin to exhibit the characteristic jigsaw-puzzle shape with lobes and sinuses. The timing of this transition appears to coincide with the differentiation of its sister cell (now a GMC) into guard cells. Is the stomatal differentiation somehow coupled with the pavement cell differentiation? If so, what is the underlying mechanism? Very recently, this question was addressed by Grones *et al.* (2020), who observed that the position of first lobe outgrowth in SLGCs is distal to the neighbouring stoma, implying the presence of signals emanating from the differentiating stoma. Indeed, observation using the auxin response reporter *DR5::NLS-Venus* revealed a gradient of auxin response in a spiral manner within each stomatal complex, with the GMC exhibiting the lowest auxin response, followed by the youngest SLGC, and the highest response in the oldest SLGC (Grones *et al.*, 2020). A previous study showed that the auxin efflux carrier PIN3 accumulates in late meristemoids transitioning to GMCs (Le *et al.*, 2014). Characterization of PIN3 localization patterns and mutant phenotypes of higher order *pin3/4/7* triple mutants led to the conclusion that timely auxin efflux from GMCs is critical for stomatal differentiation (Le *et al.*, 2014). Further analysis on the spatiotemporal dynamics of auxin efflux carriers, influx carriers and transporters (e.g. PIN3, PIN7, AUX and ABCB1) supported the hypothesis that the directed auxin flow from GMC to SLGC promotes lobe formation, driving pavement cell morphogenesis (Grones *et al.*, 2020). Thus, dynamic cell–cell auxin flow co-ordinates the differentiation of both stomata and pavement cells, and thus a deeper understanding of epidermal patterning is achieved only through characterizing the stomatal complex (stomatal precursors and SLGCs) as a unit. How the core regulators of stomatal development shape the dynamic auxin flow remains an open question.

### Stomatal development in the context of intertissue communication

While this review focused on how developing stomata and other epidermal cell types influence each other, co-ordination between stomata and underlying mesophyll and vascular tissues must also be considered. For example, EPFL9 peptide, also known as Stomagen, is secreted from internal immature mesophyll cells and promotes stomatal development via antagonizing the EPF–ERECTA/TMM receptor module in the protoderm (Kondo *et al.*, 2010; Sugano *et al.*, 2010; Shimada *et al.*, 2011; Lee *et al.*, 2015). The intertissue layer communication is not unidirectional; developing epidermis also influences mesophyll cell organization, whereby each stoma is subtended by a mesophyll air space immediately underneath. In a wheat *EPF1* (*TaEPF1*) overexpression line, the mesophyll air space was not produced underneath arrested meristemoids (Lundgren *et al.*, 2019), suggesting that mature stomata are required in the epidermal layer for mesophyll air cavity formation. Therefore, physical (e.g. air flow and the humidity gradient from open stomata) and biochemical (e.g. peptides and hormones from developing guard cells) may actively influence mesophyll development. The presence of the air cavity below stomata is not unique to arabidopsis or wheat, and the connection between mature stomata and the air cavity underneath may be universal in land plants with stomata, including the moss *Physcomitrella* (Caine *et al.*, 2020). Moreover, astomatous land plants, such as liverwort (*Marcanthia polymorpha*), develop air cavities underneath their multicellular air pores (Kohchi *et al.*, 2021). While it is not known whether the air cavity development in both stomatous and astomatous land plants involves similar physiological, biochemical and/or physical mechanisms, understanding their mechanistic bases is certainly an important future direction.

Similar intertissue layer communication probably exists between vasculature and epidermal stomata development. It is known that the epidermis immediately above major veins does not develop stomata (McKown and Bergmann, 2020; Nunes *et al.*, 2020). This architecture of excluding stomata immediately above the vasculature is likely to maximize efficient transport of photosynthates (sugar) from mesophyll cells. As one might expect, *SPCH* promoter activity is absent in the epidermis immediately above the midvein, suggesting that, like non-stomatal cell files in the hypocotyls, the mechanism to initiate *SPCH* expression is absent for this specialized epidermal region. The underlying cell–cell interactions and molecular mechanisms creating this ‘stomata-free’ zone remain unexplored, and are certainly an exciting future direction for investigation.

## CONCLUSION AND PERSPECTIVES

Historically, studies of cell type differentiation in the leaf epidermis have focused on specific cell types of interest, whether stomata, trichomes or pavement cells. The identification of key genes, signalling components and regulatory mechanisms underlying the development of stomata and other leaf cell types now enables us to explore how stomatal development occurs in the context of epidermal tissues and integrates with the development of internal mesophyll and vascular tissues. Because stomata and other tissue/cell types are interconnected for generating a functional leaf, understanding how cell–cell interaction and cellular differentiation programmes are elaborated into supra-cellular tissue and organ patterning is necessary to gain a holistic view of plant development. Recent technological advancements now enable the profiling of cellular gene expression characteristics at a single-cell resolution (e.g. scRNA-seq, scATAC-seq) (Liu *et al.*, 2020) and visualization of cell–cell interactions at high spatiotemporal resolution (Seerangan *et al.*, 2020). These integrated approaches can be harnessed to design and adjust the stomatal patterns in a holistic way to maximize plant productivity and sustenance for specific agricultural conditions in a changing climate.
